# Clinicopathological characteristics and prognosis of melanoma: A retrospective cohort study in Xinjiang, China

**DOI:** 10.1371/journal.pone.0357383

**Published:** 2026-09-09

**Authors:** Caoying Wu, Yongting Yang, Chun Wang, Yaoyuan Shen, Maimaitili Buheliqimu, Mahan Yeledan, Yuan Ding, Xiaojing Kang

**Affiliations:** 1 Xinjiang Medical University, Urumqi, Xinjiang, China; 2 Department of Dermatology and Venereology, People’s Hospital of Xinjiang Uygur Autonomous Region, Urumqi, Xinjiang, China; 3 Xinjiang Key Laboratory of Dermatology Research, Urumqi, Xinjiang, China; 4 Xinjiang Clinical Research Center for Dermatology and Venereology, Urumqi, Xinjiang, China; 5 Department of Pathology, People’s Hospital of Xinjiang Uygur Autonomous Region, Urumqi, Xinjiang, China; 6 Department of Medical Research and Translational Management, People’s Hospital of Xinjiang Uygur Autonomous Region, Urumqi, Xinjiang, China; Universita degli Studi della Campania Luigi Vanvitelli, ITALY

## Abstract

The incidence of melanoma is substantially lower in Asians than in Caucasians. This study retrospectively analyzed 355 patients in Xinjiang, China, to delineate the clinicopathological features and prognosis of melanoma in this region. The median overall survival (OS) and disease-free survival (DFS) were 78.0 and 46.0 months with 1-, 3-, and 5-year OS/DFS rates of 83.3%/68.3%/57.0% and 80.4%/59.5%/43.4%, respectively. Multivariate analysis identified advanced AJCC stage and elevated LDH as independent predictors of poorer OS. For DFS, AJCC stage, presence of metastasis, elevated LDH, and aCCI predicted worse outcomes, while adjuvant therapy was protective. Crucially, subtype-specific determinants emerged: aCCI independently predicted OS (*HR* = 18.42, *P* = 0.005) and DFS (*HR* = 4.91, *P* = 0.019) in CM, while higher BMI was associated with poorer DFS in AM (*HR* = 3.58, *P* = 0.019). These findings underscore the need for subtype-aware clinical management in this region.

## Introduction

Melanoma is the most aggressive form of skin cancer, and its incidence and mortality rates vary widely by region and ethnicity. According to GLOBOCAN 2022, approximately 325,000 new cases and 57,000 deaths occur globally each year, with a 3.1% annual increase in incidence over the past decade. Notably, age-standardized incidence rates are significantly lower in Asian populations than in Caucasian populations [1]. The United States reported 110,026 new melanoma cases in 2024, which is 18.5 times the number of new cases in China, while the mortality burden for patients in China was 8.5 times that in the United States [2].

In Caucasian populations, ultraviolet exposure is a major risk factor for melanoma [3]. However, Asian patients more frequently present with acral (39–42%) and mucosal (10–23%) melanomas, which are less associated with ultraviolet radiation [4–7]. Current evidence suggests that localized trauma, chronic mechanical stress, and persistent inflammation may contribute to the pathogenesis of acral melanoma [8–11]. Genomic analyses reveal distinct mutation patterns: *BRAF* mutations occur in only 15–25% of Asian patients (compared with 40–60% in Caucasians), while *KIT* (10–20%) and *NRAS* (15–25%) mutations are more prevalent [12,13].

Xinjiang is a large administrative region in northwest China and has the highest annual average ultraviolet intensity nationwide. The region has a population of over 25 million, with local residents exhibiting substantial variations in genetic profiles, living environments, and daily lifestyle habits. Such regional population heterogeneity may contribute to distinct clinical and pathological presentations of melanoma in this area. Using a retrospective cohort from northwest China, this study sought to summarize the clinicopathological characteristics of melanoma and identify independent prognostic factors for patient survival.

## Materials and methods

### Study design and population

This retrospective cohort study analyzed clinical records of patients with pathologically confirmed melanoma treated at the People’s Hospital of Xinjiang Uygur Autonomous Region between September 2008 and December 2025. Demographic and clinicopathological parameters included age at diagnosis, sex, medical history, primary tumor site, tumor subtype/stage, aCCI [14], baseline metastasis status, metastatic sites, and history of trauma. Pathological features, including tumor-infiltrating lymphocytes (TIL), Breslow thickness, mitotic count, and Clark level were obtained from biopsy reports, incomplete records were independently reviewed by two experienced dermatopathologists. All patients were followed up through regular outpatient visits, telephone inquiries, or electronic medical record review from the date of pathological confirmation until the unified survival cutoff date of December 30, 2025. Two primary survival endpoints were predefined: overall survival (OS) referred to the time interval from pathological diagnosis to all-cause mortality; disease-free survival (DFS) was analyzed only in patients receiving surgical resection, calculated from radical surgery to the first occurrence of local recurrence, distant metastasis or death. Right censoring was applied for all survival analyses, including two categories: administrative censoring for patients without endpoint events at the data cutoff, and loss-to-follow-up censoring for patients untraceable before any endpoint occurred. After excluding 61 patients with incomplete demographic data and 27 patients without valid follow-up data, a final cohort of 355 eligible patients was included.

### Statistical analysis

Kaplan-Meier survival curves were constructed, and group comparisons were made using the log-rank test. Cox proportional hazards models were used for univariate and multivariate analyses, with variables meeting *P* < 0.10 in univariate analysis included in the multivariate models. Statistical significance was set at *P* < 0.05 [15]. Analyses were performed using SPSS 27.0 (IBM Corp.).

### Ethical considerations

This retrospective cohort study was approved by the Ethics Committee of the People’s Hospital of Xinjiang Uygur Autonomous Region (approval ID: KY2025042203), and written informed consent was waived by the committee. Although researchers temporarily accessed participants’ personally identifiable information during data extraction, all clinical data were processed strictly in accordance with institutional ethical standards to safeguard patient confidentiality.

## Results

### Patient characteristics

A total of 355 melanoma patients were included after excluding those with incomplete demographic data (n = 61) or loss to follow-up (n = 27). The median follow-up duration was 13.0 months (range: 1.0-166.0 months). According to the AJCC 8^th^ edition: in situ 14.9% (47/355), stage I 6.7% (21/355), stage II 29.5% (93/355), stage III 24.8% (78/355), and stage IV 24.1% (76/355). Subtypes included AM (36.9%, 131/355), CM (32.7%, 116/355), mucosal melanoma (MM) (17.5%, 62/355), eye melanoma (3.4%, 12/355), and melanoma of unknown primary (MUP) (9.6%, 34/355). Histopathological examination revealed acral lentiginous melanoma (ALM) as predominant (46.6%, 115/247), followed by nodular melanoma (NM, 25.1%, 62/247), lentigo maligna melanoma (LMM, 14.2%, 35/247), and superficial spreading melanoma (SSM, 12.1%, 30/247), with 13 cases arising from nevus transformation. Among 89 genetically tested patients, 33 (37.1%) harbored driver mutations, including *BRAF* mutations in 26 (29.2%) and *NRAS* mutations in 7 (7.9%). The lungs, liver, and lymph nodes were the most common metastatic sites regardless of the primary tumor site (Table 1).

**Table 1:** Characteristics of the 355 patients in our study.

Characteristics		Total (n = 355)	CM(n = 116)	AM(n = 131)	MM(n = 62)	Eye(n = 12)	MUP(n = 34)
**Age(years)**	<60	161 (45.4)	58 (50.0)	52 (39.7)	20 (32.3)	7 (58.3)	24 (70.6)
	≥60	194 (54.6)	58 (50.0)	79 (60.3)	42 (67.7)	5 (41.7)	10 (29.4)
**Duration(months), median [IQR]** ^**a**^		12.0 [4.0,60.0]	36.0 [9.5,120.0]	24.0 [12.0,84.0]	4.0 [1.5,7.5]	4.5 [1.5,37.5]	3.0 [1.0,8.0]
**aCCI** ^**a**^	<3	194 (56.4)	68 (60.2)	59 (47.6)	32 (51.6)	7 (63.6)	28 (82.4)
	≥3	150 (43.6)	45 (39.8)	65 (52.4)	30 (48.4)	4 (36.4)	6 (17.6)
**BMI** ^**a**^	<25	174 (57.0)	58 (57.4)	61 (56.5)	29 (52.7)	8 (66.7)	18 (62.1)
	≥25	131 (43.0)	43 (42.6)	47 (43.5)	26 (47.3)	4 (33.3)	11 (37.9)
**Gender, *n* (%)**	Male	167 (47.0)	47 (40.5)	64 (48.9)	25 (40.3)	7 (58.3)	24 (70.6)
	Female	188 (53.0)	69 (59.5)	67 (51.1)	37 (59.7)	5 (41.7)	10 (29.4)
**AJCC stage,*n* (%)** ^**a**^	0	47 (14.9)	19 (17.6)	28 (22.6)	0	0	0
	I	21 (6.7)	12 (11.1)	8 (6.5)	1 (2.4)	0	0
	II	93 (29.5)	30 (27.8)	43 (34.7)	15 (35.7)	5 (55.6)	0
	III	78 (24.8)	30 (27.8)	27 (21.8)	10 (23.8)	1 (11.1)	12 (35.3)
	IV	76 (24.1)	17 (15.7)	18 (14.5)	16 (38.1)	3 (33.3)	22 (64.7)
**Ki-67, *n* (%)** ^**a**^	≤30%	118 (50.4)	42 (58.3)	41 (51.2)	15 (28.8)	7 (77.8)	13 (61.9)
	>30%	116 (49.6)	30 (41.7)	39 (48.8)	37 (71.2)	2 (22.2)	8 (38.1)
**Histology subtypes** ^**b**^	ALM	115 (47.5)	3 (2.7)	112 (83.0)	–	–	–
	NM	62 (25.6)	46 (41.4)	16 (11.9)	–	–	–
	LMM	35 (14.5)	35 (31.5)	0	–	–	–
	SSM	30 (12.4)	27 (24.3)	3 (2.2)	–	–	–
**Trauma, *n* (%)** ^**b**^	With	74 (40.4)	26 (29.9)	48 (50.0)	–	–	–
	Without	109 (59.6)	61 (70.1)	48 (50.0)	–	–	–
**Ulceration, *n* (%)** ^**b**^	With	101 (47.9)	38 (39.2)	63 (55.3)	–	–	–
	Without	110 (52.1)	59 (60.8)	51 (44.7)	–	–	–
**Breslow thickness (mm), *n* (%)** ^**b**^	≤4 mm	126 (60.9)	50 (53.2)	76 (67.3)	–	–	–
	>4mm	81 (39.1)	44 (46.8)	37 (32.7)	–	–	–
**Clark, *n* (%)** ^**b**^	I&II&III	68 (31.6)	30 (28.0)	39 (32.8)	–	–	–
	IV&V	147 (68.4)	67 (72.0)	80 (67.2)	–	–	–
**TIL, *n* (%)** ^**b**^	Absence	82 (42.1)	27 (31.4)	55 (50.2)	–	–	–
	Non-brisk	79 (40.5)	38 (44.2)	41 (37.6)	–	–	–
	Brisk	34 (17.4)	21 (24.4)	13 (11.9)	–	–	–
**Metastasis, *n* (%)** ^**a**^	With	123 (34.6)	34 (29.3)	30 (22.9)	24 (38.7)	3 (25.0)	32 (94.1)
	Without	232 (65.4)	82 (70.7)	101 (77.1)	38 (61.3)	9 (75.0)	2 (5.9)
**Metastasis site, *n* (%)** ^**b**^	Lymph node	94 (48.5)	27 (54.0)	26 (60.5)	16 (37.2)	3 (60.0)	22 (40.7)
	Lung	22 (11.3)	6 (12.0)	7 (16.3)	5 (11.6)	0	4 (7.4)
	Liver	21 (10.8)	4 (8.0)	4 (9.3)	6 (14.0)	1 (20.0)	6 (11.1)
	Bone	14 (7.2)	3 (6.0)	1 (2.3)	4 (7.5)	0	6 (11.1)
	Brain	10 (5.2)	2 (4.0)	1 (2.3)	3 (7.0)	0	5 (9.3)
	Others	33 (17.0)	8 (16.0)	4 (9.3)	9 (20.9)	1 (20.0)	11 (20.4)
**LDH(U/L)** ^**a**^	<250	213 (85.5)	78 (91.8)	84 (90.3)	37 (78.7)	5 (71.4)	9 (52.9)
	≥250	36 (14.5)	7 (8.2)	9 (9.7)	10 (21.3)	2 (28.6)	8 (47.1)
**Adjuvant therapy, *n* (%)**	With	128 (36.1)	38 (32.8)	41 (31.3)	28 (45.2)	4 (33.3)	17 (50.0)
	Without	227 (63.9)	78 (67.1)	90 (68.7)	34 (54.8)	8 (66.6)	17 (50.0)

^a^cases with missing data were excluded from the analysis; ^b^cases with missing data for CM and AM were excluded from the analysis. *AM* Acral melanoma, *CM C*utaneous melanoma, *MM* Mucosal melanoma, *MUP M*elanoma of unknown primary, *SD S*tandard deviation, *IQR* Interquartile range, *aCCI A*ge-adjusted Charlson Comorbidity Index, *BMI B*ody mass index, *AJCC* American Joint Committee on Cancer, *ALM* Acral lentiginous melanoma, *NM N*odular melanoma, *LMM L*entigo maligna melanoma, *SSM* Superficial spreading melanoma, *TIL* Tumor-infiltrating lymphocytes, *LDH* Lactate dehydrogenase.

**Fig 1:**
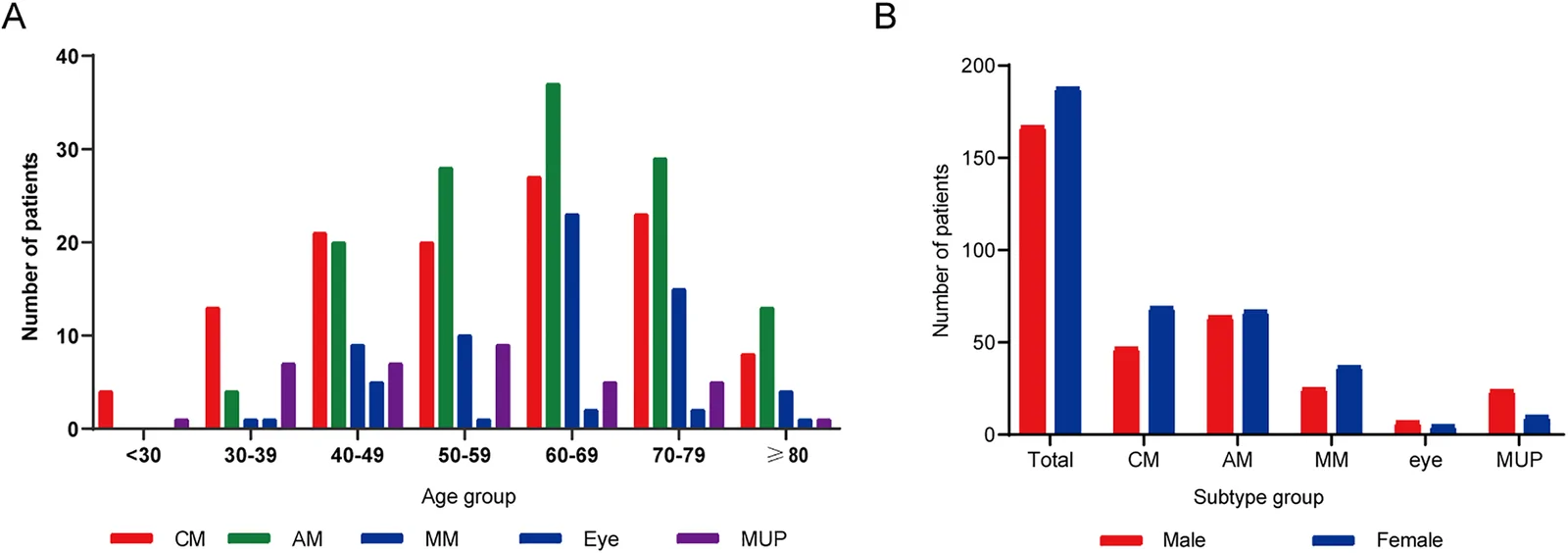
Melanoma distribution by age groups (A) and gender groups (B).

**Fig 2:**
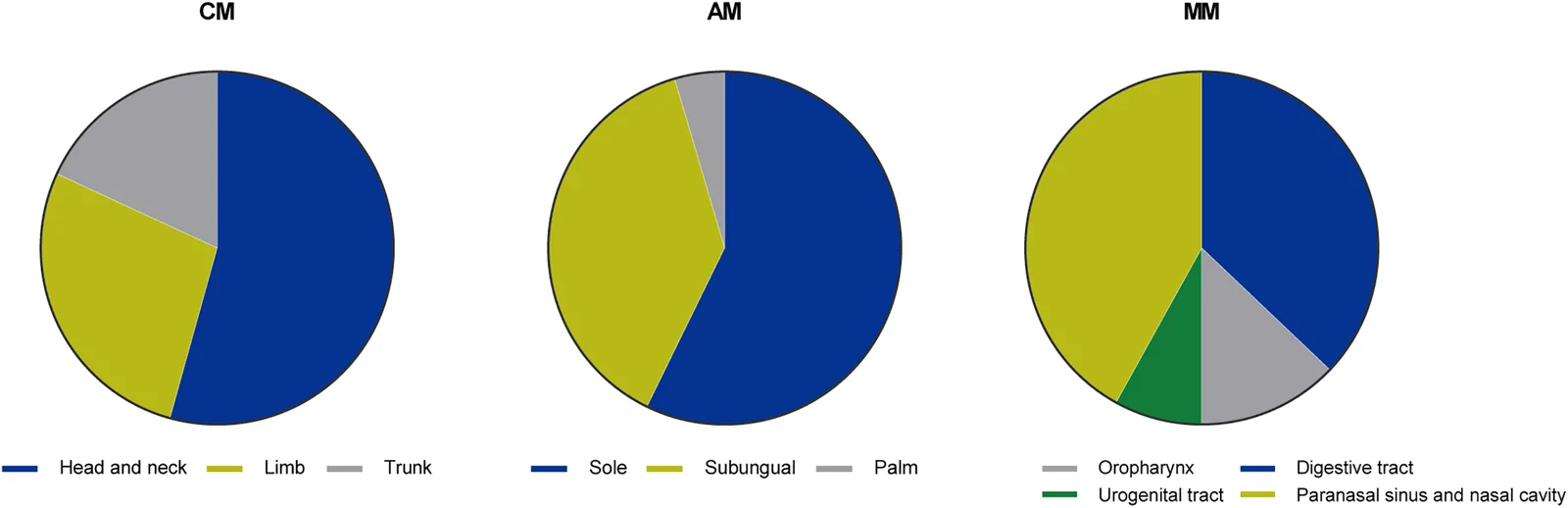
Distribution of primary tumor sites of CM, AM, and MM.

All participants were Chinese from 14 administrative divisions across China. Males comprised 47.0% (167/355), and females 53.0% (188/355), with a mean diagnosis age of 59.9 ± 14.1 years (range: 24–100). Incidence peaked at 60–69 years, while MUP showed a significantly earlier peak onset (Fig 1A). Gender distribution varied across subtypes (Fig 1B): CM, AM, and MM were more common in females (67/131, 51.1% and 37/62, 59.7%, respectively), while MUP and eye melanoma showed a male predominance. Anatomical distributions of CM, AM, and MM are detailed in Fig 2.

### Overall survival and prognostic factors

**Fig 3:**
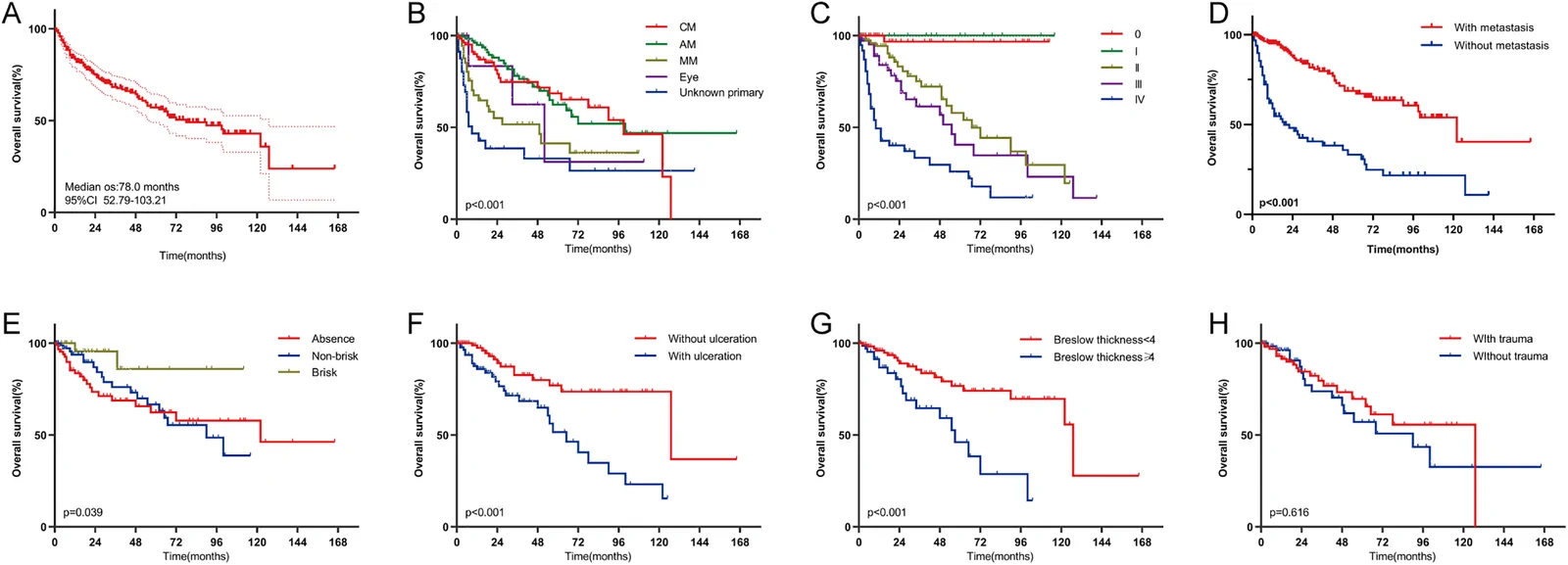
Kaplan-Meier analysis of overall survival (OS) for melanoma. OS for all patients (A), OS based on subtypes (B), AJCC stages (C), (D), TIL (E), ulceration (F), Breslow thickness (G), and trauma (H).

Among 355 patients, 96 (27.0%) died during follow-up. Median OS was 78.0 months (95% *CI*: 52.79-103.21; Fig 3A), with 1-, 3-, and 5-year survival rates of 83.3%, 68.3%, and 57.0%, respectively. Subtype analysis showed was not reached median OS for AM, while CM, MM, eye melanoma, and MUP had median OS of 99.0, 49.0, 52.0, and 9.0 months, respectively (*P* = 0.001; Fig 3B). By stage, the median OS was not reached for in situ and stage I, while stages II-IV showed 67.0, 55.0, and 10.0 months, respectively (*P* < 0.001; Fig 3C). Subgroup survival curves are displayed in Fig 3D-H.

**Fig 4:**
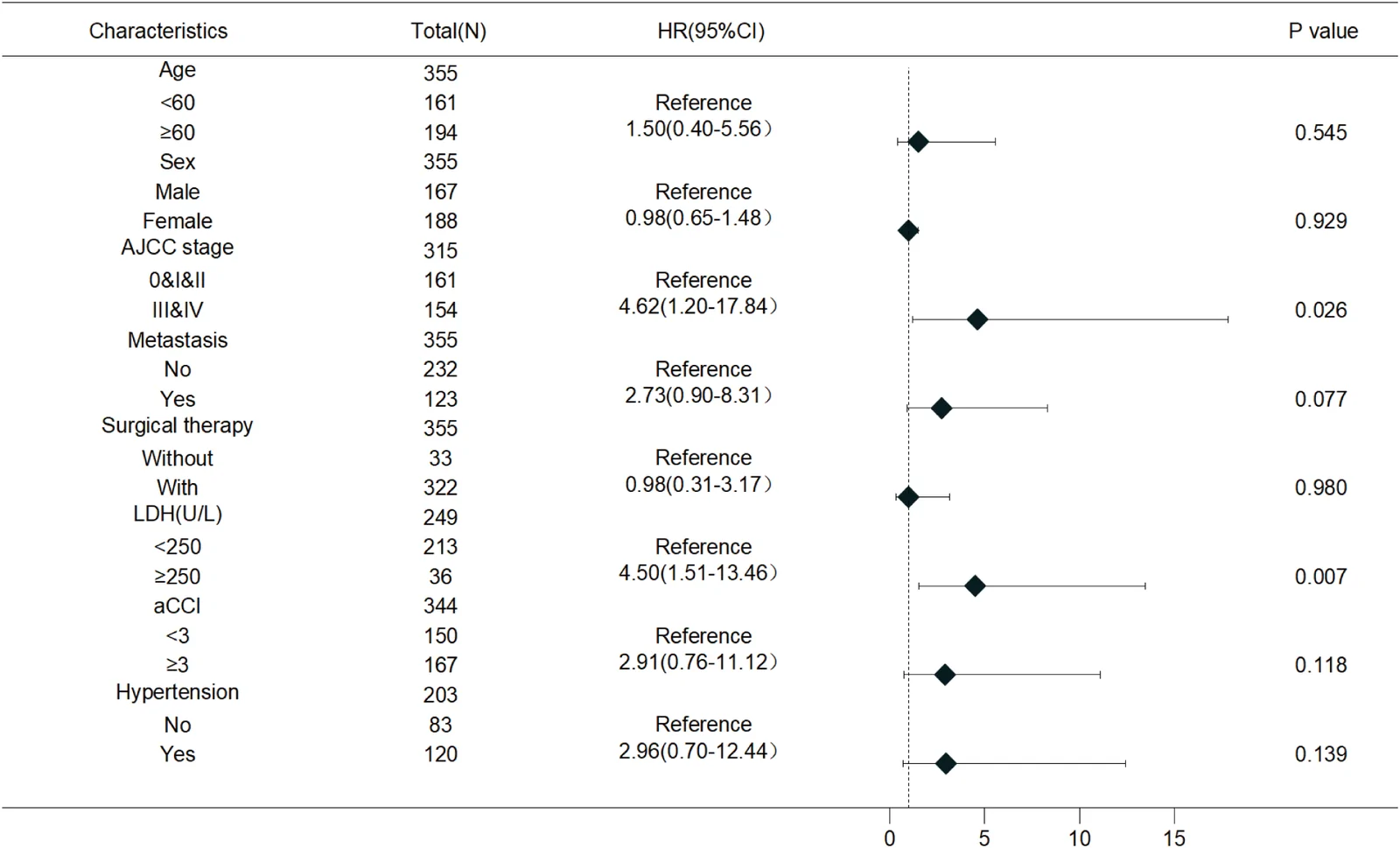
Forest plot of hazard ratios (HR) for overall survival in melanoma.

After adjustment for covariates, multivariate analysis revealed that advanced AJCC stage (III-IV) (*HR* = 4.62, 95% *CI*: 1.20-17.84, *P* = 0.026) and elevated LDH (*HR* = 4.50, 95% *CI*: 1.51-13.46, *P* = 0.005) emerged as independent risk factors for reduced OS (Fig 4 and S1 Table).

### Disease-free survival and prognostic factors

**Fig 5:**
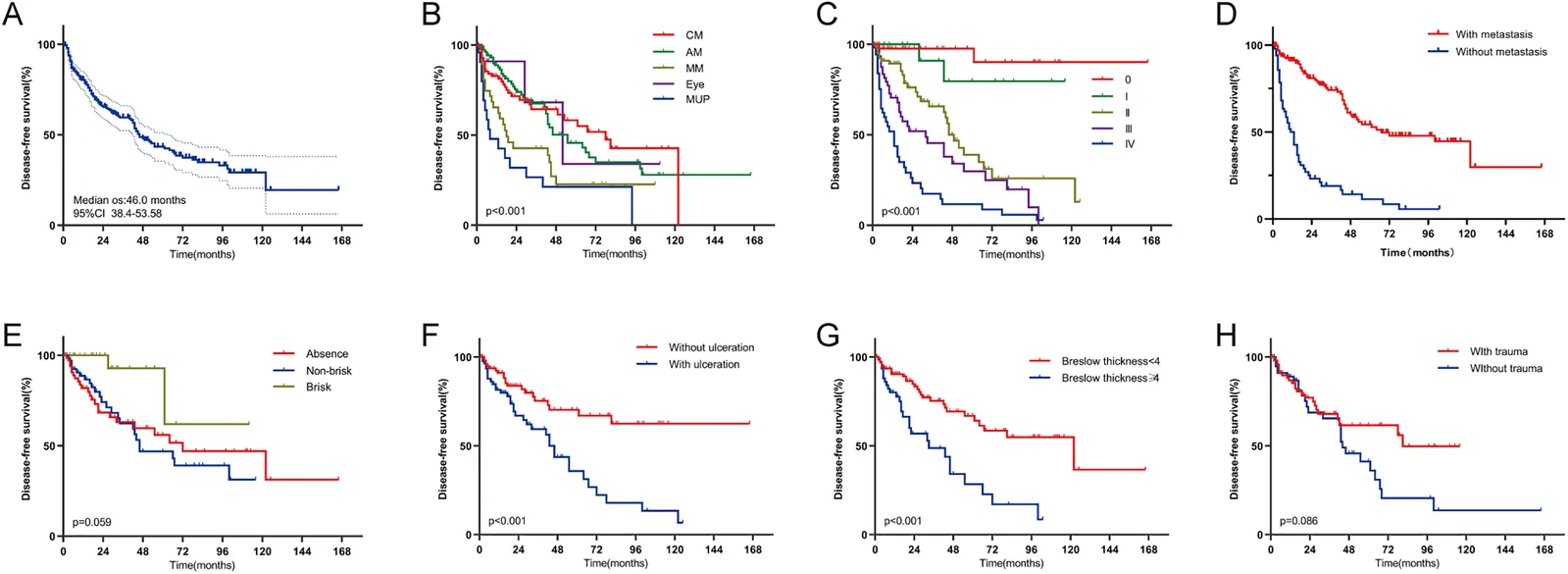
Kaplan-Meier analysis of disease-free survival (DFS) for melanoma. DFS for all surgical patients (A), DFS based on subtypes(B), AJCC stages(C), (D), TIL (E), ulceration(F), Breslow thickness(G), and trauma(H).

Of the 322 patients who underwent surgical treatment, 119 (37.0%) experienced recurrence or death during follow-up, with a median DFS of 46.0 months (95% *CI*: 38.43-53.58) (Fig 5A). The recurrence-free rates at 1 year, 3 years, and 5 years were 80.4%, 59.5%, and 43.4%, respectively. By subtype, the median DFS for CM, AM, MM, eye, and MUP was 78.0, 55.0, 18.0, 52.0, and 8.0 months, respectively (*P* < 0.001) (Fig 5B). The median DFS was not reached for in situ and stage I patients, while stage II-IV patients showed 49.0, 32.0, and 13.0 months, respectively(*P* < 0.001) (Fig 5C). Survival curves for these subgroups are shown in Fig 5D–H.

**Fig 6:**
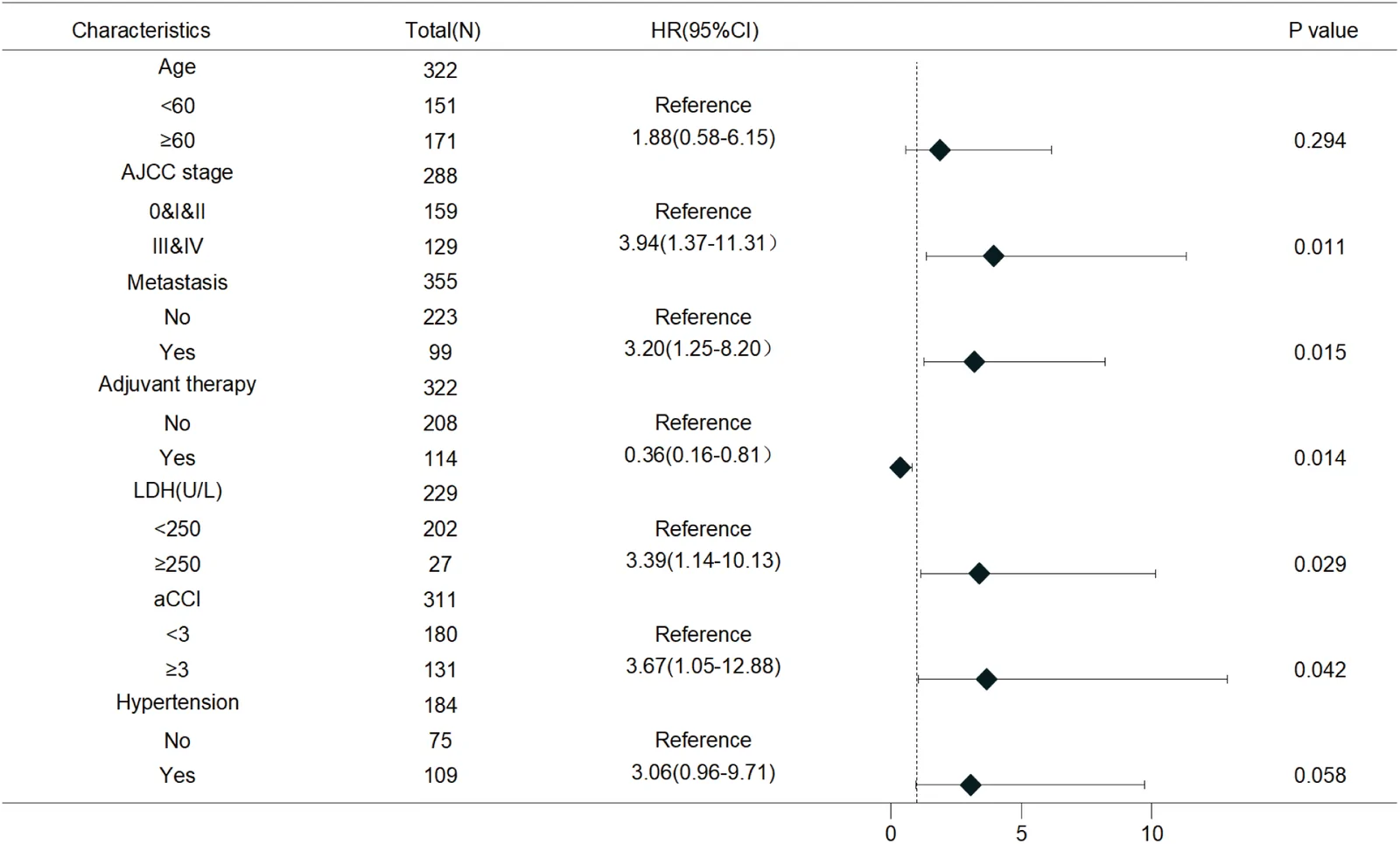
Forest plot of hazard ratios(HR) for disease-free survival in melanoma.

Multivariate analysis identified that AJCC stage III/IV (*HR* = 3.94, 95% *CI*: 1.37-11.31, *P* = 0.011), presence of metastasis (*HR* = 3.20, 95% *CI*: 1.25-8.20, *P* = 0.015), LDH ≥ 250 U/L (*HR* = 3.40, 95% *CI*: 1.14-10.13, *P* = 0.029), and aCCI ≥ 3 (*HR* = 3.67, 95% *CI*: 1.05-12.88, *P* = 0.042) were identified as independent risk factors for DFS, with adjuvant therapy (*HR* = 0.36, 95% *CI*:0.16-0.81, *P* = 0.014),while adjuvant therapy conferring significant protection (Fig 6 and S2 Table).

### Prognostic factors for subtypes

Multivariable analyses identified metastasis (*HR* = 9.39, 95% *CI*: 1.18-74.94, *P* = 0.035), ulceration (*HR* = 4.50, 95% *CI*: 1.15-17.66, *P* = 0.031), and aCCI ≥ 3 (*HR* = 18.42, 95% *CI*: 2.36-143.63, *P* = 0.005) as independent predictors of OS in CM while metastasis (*HR* = 11.32, 95% *CI*: 2.06-62.16, *P* = 0.005) and aCCI ≥ 3 (*HR* = 4.91, 95% *CI*: 1.30-18.53, *P* = 0.019) were associated with impaired DFS (S3 and S4 Table). For AM, Clark IV/V (*HR* = 7.25, 95% *CI*: 1.37-38.44, *P* = 0.020), metastasis (*HR* = 6.17, 95% *CI*: 1.23-30.99, *P* = 0.027), and Breslow thickness >4 mm (*HR* = 6.02, 95% *CI*: 1.15-31.59, *P* = 0.034) independently predicted worse OS, whereas Clark IV-V (*HR* = 18.51, *CI*: 3.24-105.76, *P* = 0.001) and BMI ≥ 25 (*HR* = 3.58, *CI*: 1.23-10.42, *P* = 0.019) independently impacted DFS (S5 and S6 Table). In MM, LDH ≥ 250 U/L (*HR* = 7.05, 95% *CI*: 1.41-35.31, *P* = 0.018) independently predicted worse OS, while age ≥ 65 years (*HR* = 6.33, 95% *CI*: 2.15-18.71, *P* = 0.001) and LDH ≥ 250 U/L (*HR* = 4.76, 95% *CI*: 1.15-19.72, *P* = 0.034) were significantly associated with reduced DFS (S7 and S8 Table).

Univariate analysis suggested that visceral metastasis (*HR* = 6.97, 95% *CI*: 1.53–31.68, *P* = 0.012), LDH ≥ 250 U/L (*HR* = 17.56, 95% *CI*: 2.06–149.89, *P* = 0.009), and aCCI ≥ 3 (*HR* = 4.18, 95% *C*I: 1.42–12.29, *P* = 0.009) are associated with worse OS in MUP, and visceral metastasis compromised DFS (*HR* = 6.94, 95% *CI*: 1.48–32.61, *P* = 0.014) (S9 and S10 Table). Due to the limited cohort size of eye melanoma, Cox proportional hazards regression analysis was not conducted to avoid overfitting. S11 Table presents the discriminative ability (C-index), goodness-of-fit (AIC/BIC), and proportional hazards assumption test results of the Cox regression models across all subgroups.

## Discussion

Xinjiang is China’s largest provincial-level administrative region, accounting for approximately one-sixth of the country’s total land area. Limited data are available on melanoma in Xinjiang. As the largest tertiary referral center in Xinjiang, our hospital cares for the majority of melanoma patients from all 14 administrative divisions of the region, offering a representative cohort to investigate the distinct epidemiological distribution and prognostic features of melanoma in northwest China.

Our findings reveal several distinctive features of melanoma in Xinjiang. The age distribution at diagnosis is consistent with recent data from both Western and Asian populations [4,16,17]. According to the Global Burden of Disease (GBD) [18], melanoma incidence demonstrates a sex-specific pattern: it is more prevalent in males among younger individuals (<60 years), whereas females show higher rates in older adults (≥60 years). In contrast, our cohort revealed no age-dependent sex disparity, with a female predominance observed across the two groups.

Consistent with findings from prior Asian cohort studies, AM (36.9%) was the predominant subtype within our study [19]. Notably, CM ranked the second most prevalent subtype (32.7%), surpassing MM, which was previously reported as the second most common subtype in Asian cohorts. The proportion of LMM in this cohort was 14.2%, which is substantially higher than the rates documented in other Asian populations (China: 0.6% [5], Korea: 3.3% [19], Japan: 8.1% [6]) and approaching the 15.7% prevalence in the US population documented in the SEER database [20]. This observation may be attributed to the region’s high-intensity ultraviolet radiation exposure and distinct genetic predispositions, warranting further mechanistic investigation.

Consistent with previous epidemiological findings [4,5], our study demonstrated that MUP predominantly manifests at a younger age, with lymph nodes as the most common initial metastatic site. It is noteworthy that the MUP prevalence (9.6%) exceeded SEER database rates [21], potentially owing to higher proportions of AM/MM subtypes prone to occult primaries in our study.

The mutational landscape of melanoma exhibits profound ethnic heterogeneity. In Caucasian populations, approximately 40−60% of melanomas harbor *BRAF* mutations, a molecular signature tightly linked to UV-driven carcinogenesis and prognosis [22–24]. Our cohort showed an overall *BRAF* mutation frequency of 29.2%, consistent with previous domestic reports [13,25]. Intriguingly, CM cases in our cohort showed a *BRAF* mutation rate (52.5%) comparable to that in Caucasians, whereas AM displayed a markedly lower prevalence (8.1%). This subtype heterogeneity likely contributes to the poorer prognosis observed in Chinese melanoma patients, as it restricts therapeutic opportunities for BRAF inhibitors in this population. However, no survival difference was observed between CM and AM in our cohort.

MM is widely recognized for its poor prognosis, with 5-year OS rates below 30% in many studies [26–28]. Our findings support this observation: MM cases in our cohort were diagnosed at later stages and had significantly shorter disease duration (4.0 months) compared with CM (34.0 months) and AM (24.0 months). This is likely attributable to MM’s subtle and concealed clinical presentation, which contributes to delayed diagnosis.

Prognostic factors varied among melanoma subtypes. Previous research has shown that Chinese AM patients tend to have greater Breslow thickness and higher ulceration rates [29]. Additionally, AM is associated with less TIL infiltration and a higher history of trauma [30,31]. Our results support these findings. Furthermore, ulceration and Breslow thickness were significantly associated with both OS and DFS in Kaplan-Meier analyses. Although higher TIL levels were associated with improved OS, they had no significant effect on DFS. Interestingly, trauma history did not correlate with either OS or DFS in multivariate models.

AJCC Stage and LDH emerged as independent OS/DFS predictors across subtypes. Previous studies have reported that comorbidities are closely associated with disease incidence, prognosis, and immune-related adverse events [32,33]. Our study revealed that aCCI was an independent risk factor for OS and DFS in CM, but had no prognostic impact on AM. Notably, comorbidity prevalence is reportedly higher in the Xinjiang region [34,35]. With the advent of an aging population, the prognostic implications of comorbidities in melanoma warrant increased attention. Additionally, we identified BMI as an independent risk factor for DFS of AM, with higher BMI correlating with an increased recurrence risk. Although prior studies suggested BMI as a protective factor for OS in AM [36], its association with DFS remains unreported. Emerging research highlights the “obesity paradox”, although obesity promotes immune senescence, tumor progression, and PD-1-mediated T-cell dysfunction, it exhibits a paradoxical association with enhanced PD-1/PD-L1 blockade efficacy [37] and may reduce melanoma-specific mortality [38]. However, in AM, obesity may play distinct prognostic roles through mechanical stress (particularly in plantar-predominant lesions), necessitating further investigation.

This study has several limitations. As a single-center retrospective analysis conducted over a long time span, there is potential for bias in data collection and evolving treatment approaches. Nonetheless, data completeness was carefully maintained. The relatively small sample size also limited statistical power, particularly for rare subtypes like ocular melanoma, for which multivariate analysis was not feasible. Stratified subtype multivariate Cox analyses yielded extremely wide confidence intervals for several hazard ratios due to limited subgroup sample sizes and sparse survival events. Readers should not interpret the exact numerical values of these effect sizes rigidly; greater weight should be placed on whether the association is statistically significant and the direction of prognostic impact, rather than literal HR magnitudes. Despite these limitations, our study provides valuable insights into the epidemiology and prognosis of melanoma in a geographically distinct population of northwest China. We are currently working to establish a multicenter research network in Xinjiang to validate and expand upon these findings.

## Conclusions

In summary, this study highlights the unique clinicopathological features and prognostic factors of melanoma in northwest China. Differences in anatomical distribution, histological subtypes, mutation profiles, and survival outcomes distinguish this population from both Caucasian cohorts and other regions in China. Subtype-specific prognostic indicators—such as aCCI in CM and BMI in AM—underscore the need for individualized clinical strategies and provide a foundation for future multicenter research and public health policy planning in western China.

## Supporting information

S1 Table Univariate and multivariate analysis of risk factors associated with OS. (DOCX)

S2 Table Univariate and multivariate analysis of risk factors associated with DFS. (DOCX)

S3 Table Univariate and multivariate analysis of risk factors for OS in CM. (DOCX)

S4 Table Univariate and multivariate analysis of risk factors for DFS in CM. (DOCX)

S5 Table Univariate and multivariate analysis of risk factors for OS in AM. (DOCX)

S6 Table Univariate and multivariate analysis of risk factors for DFS in AM. (DOCX)

S7 Table Univariate and multivariate analysis of risk factors for OS in MM. (DOCX)

S8 Table Univariate and multivariate analysis of risk factors for DFS in MM. (DOCX)

S9 Table Univariate and multivariate analysis of risk factors for OS in MUP. (DOCX)

S10 Table Univariate and multivariate analysis of risk factors for DFS in MUP. (DOCX)

S11 Table Cox regression model performance evaluation across all subgroups. (DOCX)

S1 File Supplementary materials 2. (XLSX)
