# Supplementary material for: Clinicopathological characteristics and prognosis of melanoma: A retrospective cohort study in Xinjiang, China
Source: PLoS One. 2026 Sep 9;21(9):e0357383. doi: 10.1371/journal.pone.0357383 (PMC13557341; doi:10.1371/journal.pone.0357383)
Supplement: Supplementary file 1 [file pone.0357383.s001.docx]

S1 Table. Univariate and multivariate analysis of risk factors associated with OS.

| Characteristics | Univariate analysis | | | Multivariate analysis | | |
| --- | --- | --- | --- | --- | --- | --- |
|  | HR | 95%CI | *p* value | HR | 95%CI | p value |
| Age |  |  |  |  |  |  |
| <60 |  | Reference |  |  | Reference |  |
| ≥60 | 1.66 | 1.10 – 2.51 | 0.016 | 1.50 | 0.40-5.56 | 0.545 |
| Gender |  |  |  |  |  |  |
| Female |  | Reference |  |  | Reference |  |
| Male | 1.40 | 0.94– 2.09 | 0.098 | 0.98 | 0.65-1.48 | 0.929 |
| AJCC stage |  |  |  |  |  |  |
| 0&I&II |  | Reference |  |  | Reference |  |
| III&IV | 4.44 | 2.76-7.15 | < 0.001 | 4.62 | 1.20-17.84 | 0.026 |
| Metastasis |  |  |  |  |  |  |
| Without |  | Reference |  |  | Reference |  |
| With | 4.02 | 2.67-6.05 | < 0.001 | 2.73 | 0.90-8.31 | 0.077 |
| Surgical therapy |  |  |  |  |  |  |
| Without |  | Reference |  |  | Reference |  |
| With | 0.30 | 0.17-0.52 | < 0.001 | 0.98 | 0.31-3.17 | 0.980 |
| Adjuvant therapy |  |  |  |  |  |  |
| Without |  | Reference |  |  |  |  |
| With | 0.77 | 0.51-1.15 | 0.198 |  |  |  |
| LDH, U/L |  |  |  |  |  |  |
| <250 |  | Reference |  |  | Reference |  |
| ≥250 | 4.06 | 2.30-7.15 | < 0.001 | 4.50 | 1.51-13.46 | 0.007 |
| BMI |  |  |  |  |  |  |
| <25 |  | Reference |  |  |  |  |
| ≥25 | 0.99 | 0.64-1.53 | 0.947 |  |  |  |
| aCCI |  |  |  |  |  |  |
| <3 |  | Reference |  |  | Reference |  |
| ≥3 | 1.67 | 1.07 - 2.42 | 0.024 | 2.91 | 0.76-11.12 | 0.118 |
| Diabetes |  |  |  |  |  |  |
| Without |  | Reference |  |  |  |  |
| With | 1.39 | 0.77-2.52 | 0.279 |  |  |  |
| Hypertension |  |  |  |  |  |  |
| Without |  | Reference |  |  | Reference |  |
| With | 2.60 | 1.02-6.65 | 0.046 | 2.96 | 0.70-12.44 | 0.139 |

*OS O*verall survival, *HR* Hazard ratio, *CI* Confidence interval, *AJCC* American Joint Committee on Cancer, *LDH* Lactate dehydrogenase, *BMI* Body mass index, *aCCI* Age-adjusted Charlson Comorbidity Index
