# Supplementary material for: Clinicopathological characteristics and prognosis of melanoma: A retrospective cohort study in Xinjiang, China
Source: PLoS One. 2026 Sep 9;21(9):e0357383. doi: 10.1371/journal.pone.0357383 (PMC13557341; doi:10.1371/journal.pone.0357383)
Supplement: Supplementary file 2 [file pone.0357383.s002.docx]

S2 Table. Univariate and multivariate analysis of risk factors associated with DFS.

| Characteristics | Univariate analysis | | | Multivariate analysis | | |
| --- | --- | --- | --- | --- | --- | --- |
|  | HR | 95%CI | p value | HR | 95%CI | p value |
| Age |  |  |  |  |  |  |
| <60 |  | Reference |  |  | Reference |  |
| ≥60 | 1.47 | 1.01-2.14 | 0.045 | 1.88 | 0.58-6.15 | 0.294 |
| Gender |  |  |  |  |  |  |
| Female |  | Reference |  |  |  |  |
| Male | 1.34 | 0.92-1.94 | 0.124 |  |  |  |
| AJCC stage |  |  |  |  |  |  |
| 0&I&II |  | Reference |  |  | Reference |  |
| III&IV | 4.46 | 2.90-6.85 | <0.001 | 3.94 | 1.37-11.31 | 0.011 |
| Metastasis |  |  |  |  |  |  |
| Without |  | Reference |  |  | Reference |  |
| With | 4.12 | 2.83-6.01 | <0.001 | 3.20 | 1.25-8.20 | 0.015 |
| Adjuvant therapy |  |  |  |  |  |  |
| Without |  | Reference |  |  | Reference |  |
| With | 0.60 | 0.42-0.86 | 0.005 | 0.36 | 0.16-0.81 | 0.014 |
| LDH，U/L |  |  |  |  |  |  |
| <250 |  | Reference |  |  | Reference |  |
| ≥250 | 2.32 | 1.30-4.15 | 0.004 | 3.39 | 1.14-10.13 | 0.029 |
| BMI |  |  |  |  |  |  |
| <25 |  | Reference |  |  |  |  |
| ≥25 | 0.93 | 0.62-1.40 | 0.737 |  |  |  |
| aCCI |  |  |  |  |  |  |
| <3 |  | Reference |  |  | Reference |  |
| ≥3 | 1.60 | 1.09-2.34 | 0.016 | 3.67 | 1.05-12.88 | 0.042 |
| Diabetes |  |  |  |  |  |  |
| Without |  | Reference |  |  |  |  |
| With | 1.18 | 0.67-2.08 | 0.570 |  |  |  |
| Hypertension |  |  |  |  |  |  |
| Without |  | Reference |  |  | Reference |  |
| With | 2.23 | 1.00-4.99 | 0.051 | 0.53 | 0.16-1.73 | 0.294 |

*DFS* Disease-free survival, *HR* Hazard ratio, *CI* Confidence interval, *AJCC* American Joint Committee on Cancer, *LDH* Lactate dehydrogenase, *BMI* Body mass index, *aCCI* Age-adjusted Charlson Comorbidity Index
