# Supplementary material for: Clinicopathological characteristics and prognosis of melanoma: A retrospective cohort study in Xinjiang, China
Source: PLoS One. 2026 Sep 9;21(9):e0357383. doi: 10.1371/journal.pone.0357383 (PMC13557341; doi:10.1371/journal.pone.0357383)
Supplement: Supplementary file 3 [file pone.0357383.s003.docx]

S3 Table. Univariate and multivariate analysis of risk factors for OS in CM.

| Characteristics | Univariate analysis | | | Multivariate analysis | | |
| --- | --- | --- | --- | --- | --- | --- |
|  | HR | 95%CI | p value | HR | 95%CI | p value |
| Age |  |  |  |  |  |  |
| <60 |  | Reference |  |  | Reference |  |
| ≥60 | 2.37 | 1.00 – 5.65 | 0.051 | 2.61 | 0.06-2.68 | 0.334 |
| Gender |  |  |  |  |  |  |
| Female |  | Reference |  |  |  |  |
| Male | 1.66 | 0.73– 3.81 | 0.230 |  |  |  |
| AJCC stage |  |  |  |  |  |  |
| Tis&I&II |  | Reference |  |  | Reference |  |
| III&IV | 4.96 | 1.99-12.36 | 0.001 | 1.73 | 0.22-13.35 | 0.602 |
| Metastasis |  |  |  |  |  |  |
| Without |  | Reference |  |  | Reference |  |
| With | 4.40 | 1.93-10.07 | < 0.001 | 9.39 | 1.18-74.94 | 0.035 |
| Clark |  |  |  |  |  |  |
| I&II&III |  | Reference |  |  |  |  |
| IV&V | 2.96 | 0.84-10.40 | 0.092 |  |  |  |
| Breslow thickness |  |  |  |  |  |  |
| ≤4 |  | Reference |  |  | Reference |  |
| >4 | 5.55 | 1.84-16.76 | 0.002 | 2.75 | 0.42-18.10 | 0.292 |
| Ulceration |  |  |  |  |  |  |
| Without |  | Reference |  |  | Reference |  |
| With | 3.39 | 1.30-8.80 | 0.012 | 4.50 | 1.15-17.66 | 0.031 |
| Surgical therapy |  |  |  |  |  |  |
| Without |  | Reference |  |  | Reference |  |
| With | 0.09 | 0.02-0.43 | 0.002 | 0.18 | 0.24-127.06 | 0.283 |
| Adjuvant therapy |  |  |  |  |  |  |
| Without |  | Reference |  |  |  |  |
| With | 1.07 | 0.47-2.46 | 0.868 |  |  |  |
| LDH，U/L |  |  |  |  |  |  |
| <250 |  | Reference |  |  |  |  |
| ≥250 | 2.41 | 0.67-8.40 | 0.169 |  |  |  |
| BMI |  |  |  |  |  |  |
| <25 |  | Reference |  |  |  |  |
| ≥25 | 0.47 | 0.18-1.22 | 0.119 |  |  |  |
| aCCI |  |  |  |  |  |  |
| <3 |  | Reference |  |  | Reference |  |
| ≥3 | 4.47 | 1.80-11.08 | 0.001 | 18.42 | 2.36-143.63 | 0.005 |

*OS O*verall survival, *CM* Cutaneous melanoma, *HR* Hazard ratio, *CI* Confidence interval, *AJCC* American Joint Committee on Cancer, *LDH* Lactate dehydrogenase, *BMI* Body mass index, *aCCI* Age-adjusted Charlson Comorbidity Index
