# Supplementary material for: Clinicopathological characteristics and prognosis of melanoma: A retrospective cohort study in Xinjiang, China
Source: PLoS One. 2026 Sep 9;21(9):e0357383. doi: 10.1371/journal.pone.0357383 (PMC13557341; doi:10.1371/journal.pone.0357383)
Supplement: Supplementary file 4 [file pone.0357383.s004.docx]

S4 Table. Univariate and multivariate analysis of risk factors for DFS in CM.

| Characteristics | Univariate analysis | | | Multivariate analysis | | |
| --- | --- | --- | --- | --- | --- | --- |
|  | HR | 95%CI | p value | HR | 95%CI | p value |
| Age |  |  |  |  |  |  |
| <60 |  | Reference |  |  |  |  |
| ≥60 | 1.56 | 0.77-3.14 | 0.219 |  |  |  |
| Gender |  |  |  |  |  |  |
| Female |  | Reference |  |  |  |  |
| Male | 1.49 | 0.74-3.04 | 0.268 |  |  |  |
| AJCC stage |  |  |  |  |  |  |
| Tis&I&II |  | Reference |  |  | Reference |  |
| III&IV | 5.34 | 2.40-11.87 | < 0.001 | 1.08 | 0.15-5.91 | 0.938 |
| Metastasis |  |  |  |  |  |  |
| Without |  | Reference |  |  | Reference |  |
| With | 4.72 | 2.32-9.62 | < 0.001 | 11.32 | 2.06-62.16 | 0.005 |
| Clark level |  |  |  |  |  |  |
| I&II&III |  | Reference |  |  | Reference |  |
| IV&V | 3.37 | 1.15-9.88 | 0.027 | 2.23 | 0.05-4.12 | 0.479 |
| Breslow thickness |  |  |  |  |  |  |
| ≤4 |  | Reference |  |  | Reference |  |
| >4 | 4.37 | 1.80-10.55 | 0.001 | 4.36 | 0.58-32.96 | 0.154 |
| Ulceration |  |  |  |  |  |  |
| Without |  | Reference |  |  | Reference |  |
| With | 2.51 | 1.10-5.69 | 0.028 | 2.00 | 0.55-7.25 | 0.291 |
| Adjuvant therapy |  |  |  |  |  |  |
| Without |  | Reference |  |  |  |  |
| With | 1.47 | 0.73-2.96 | 0.282 |  |  |  |
| LDH，U/L |  |  |  |  |  |  |
| <250 |  | Reference |  |  |  |  |
| ≥250 | 1.69 | 0.50-5.74 | 0.400 |  |  |  |
| BMI |  |  |  |  |  |  |
| <25 |  | Reference |  |  | Reference |  |
| ≥25 | 0.43 | 0.19-0.99 | 0.047 | 0.33 | 0.08-1.29 | 0.111 |
| aCCI |  |  |  |  |  |  |
| <3 |  | Reference |  |  | Reference |  |
| ≥3 | 3.35 | 1.56-7.19 | 0.002 | 4.91 | 1.30-18.53 | 0.019 |

*DFS* Disease-free survival, *CM* Cutaneous melanoma, *HR* Hazard ratio, *CI* Confidence interval, *AJCC* American Joint Committee on Cancer, *LDH* Lactate dehydrogenase, *BMI* Body mass index, *aCCI* Age-adjusted Charlson Comorbidity Index
