# Supplementary material for: Clinicopathological characteristics and prognosis of melanoma: A retrospective cohort study in Xinjiang, China
Source: PLoS One. 2026 Sep 9;21(9):e0357383. doi: 10.1371/journal.pone.0357383 (PMC13557341; doi:10.1371/journal.pone.0357383)
Supplement: Supplementary file 5 [file pone.0357383.s005.docx]

S5 Table. Univariate and multivariate analysis of risk factors for OS in AM.

| Characteristics | Univariate analysis | | | Multivariate analysis | | |
| --- | --- | --- | --- | --- | --- | --- |
|  | HR | 95%CI | p value | HR | 95%CI | p value |
| Age |  |  |  |  |  |  |
| <60 |  | Reference |  |  |  |  |
| ≥60 | 1.50 | 0.68-3.28 | 0.314 |  |  |  |
| Gender |  |  |  |  |  |  |
| Female |  | Reference |  |  |  |  |
| Male | 1.45 | 0.68– 3.08 | 0.338 |  |  |  |
| AJCC stage |  |  |  |  |  |  |
| 0&I&II |  | Reference |  |  | Reference |  |
| III&IV | 4.55 | 1.97-10.43 | <0.001 | 5.25 | 0.65-42.22 | 0.119 |
| Metastasis |  |  |  |  |  |  |
| Without |  | Reference |  |  | Reference |  |
| With | 4.49 | 2.06-9.79 | <0.001 | 6.17 | 1.23-30.99 | 0.027 |
| Clark level |  |  |  |  |  |  |
| I&II&III |  | Reference |  |  | Reference |  |
| IV&V | 8.87 | 2.04-38.48 | 0.004 | 7.25 | 1.37-38.44 | 0.020 |
| Breslow thickness |  |  |  |  |  |  |
| ≤4 |  | Reference |  |  | Reference |  |
| >4 | 2.12 | 0.88-5.08 | 0.093 | 6.02 | 1.15-31.59 | 0.034 |
| Ulceration |  |  |  |  |  |  |
| Without |  | Reference |  |  | Reference |  |
| With | 3.31 | 1.28-8.53 | 0.013 | 1.31 | 0.35-4.88 | 0.689 |
| Surgical therapy |  |  |  |  |  |  |
| Without |  | Reference |  |  |  |  |
| With | 0.26 | 0.03-1.98 | 0.194 |  |  |  |
| Adjuvant therapy |  |  |  |  |  |  |
| Without |  | Reference |  |  |  |  |
| With | 1.60 | 0.74-3.47 | 0.237 |  |  |  |
| LDH，U/L |  |  |  |  |  |  |
| <250 |  | Reference |  |  |  |  |
| ≥250 | 1.19 | 0.11-6.41 | 0.867 |  |  |  |
| BMI |  |  |  |  |  |  |
| <25 |  | Reference |  |  |  |  |
| ≥25 | 1.82 | 0.75-4.40 | 0.184 |  |  |  |
| aCCI |  |  |  |  |  |  |
| <3 |  | Reference |  |  |  |  |
| ≥3 | 1.22 | 0.56-2.65 | 0.618 |  |  |  |

*OS* Overall survival, *AM* Acral melanoma, *HR* Hazard ratio, *CI* Confidence interval, *AJCC* American Joint Committee on Cancer, *LDH* Lactate dehydrogenase, *BMI* Body mass index, *aCCI* Age-adjusted Charlson Comorbidity Index
