# Supplementary material for: Clinicopathological characteristics and prognosis of melanoma: A retrospective cohort study in Xinjiang, China
Source: PLoS One. 2026 Sep 9;21(9):e0357383. doi: 10.1371/journal.pone.0357383 (PMC13557341; doi:10.1371/journal.pone.0357383)
Supplement: Supplementary file 6 [file pone.0357383.s006.docx]

S6 Table. Univariate and multivariate analysis of risk factors for DFS in AM.

| Characteristics | Univariate analysis | | | Multivariate analysis | | |
| --- | --- | --- | --- | --- | --- | --- |
|  | HR | 95%CI | p value | HR | 95%CI | p value |
| Age |  |  |  |  |  |  |
| <60 |  | Reference |  |  |  |  |
| ≥60 | 1.46 | 0.79-2.70 | 0.229 |  |  |  |
| Gender |  |  |  |  |  |  |
| Female |  | Reference |  |  |  |  |
| Male | 1.32 | 0.73-2.41 | 0.358 |  |  |  |
| AJCC stage |  |  |  |  |  |  |
| 0&I&II |  | Reference |  |  | Reference |  |
| III&IV | 4.80 | 2.55-9.04 | <0.001 | 6.67 | 1.35-32.84 | 0.020 |
| Metastasis |  |  |  |  |  |  |
| Without |  | Reference |  |  | Reference |  |
| With | 4.67 | 2.54-8.59 | <0.001 | 24.70 | 4.39-138.84 | <0.001 |
| Clark level |  |  |  |  |  |  |
| I&II&III |  | Reference |  |  | Reference |  |
| IV&V | 16.01 | 3.78-67.88 | <0.001 | 18.51 | 3.24-105.76 | 0.001 |
| Breslow thickness |  |  |  |  |  |  |
| ≤4 |  | Reference |  |  | Reference |  |
| >4 | 2.02 | 1.00-4.09 | 0.049 | 3.03 | 0.09-1.15 | 0.082 |
| Ulceration |  |  |  |  |  |  |
| Without |  | Reference |  |  | Reference |  |
| With | 2.97 | 1.40-6.31 | 0.005 | 2.48 | 0.13-1.30 | 0.127 |
| Adjuvant therapy |  |  |  |  |  |  |
| Without |  | Reference |  |  | Reference |  |
| With | 0.42 | 0.23-0.78 | 0.006 | 0.15 | 0.05-0.49 | 0.001 |
| LDH，U/L |  |  |  |  |  |  |
| <250 |  | Reference |  |  |  |  |
| ≥250 | 1.39 | 0.42-4.64 | 0.591 |  |  |  |
| BMI |  |  |  |  |  |  |
| <25 |  | Reference |  |  | Reference |  |
| ≥25 | 2.00 | 0.98-4.07 | 0.055 | 3.58 | 1.23-10.42 | 0.019 |
| aCCI |  |  |  |  |  |  |
| <3 |  | Reference |  |  |  |  |
| ≥3 | 1.17 | 0.63-2.17 | 0.612 |  |  |  |

*DFS* Disease-free survival, *AM* Acral melanoma, *HR* Hazard ratio, *CI* Confidence interval, *AJCC* American Joint Committee on Cancer, *LDH* Lactate dehydrogenase, *BMI* Body mass index, *aCCI* Age-adjusted Charlson Comorbidity Index
