# Supplementary material for: Clinicopathological characteristics and prognosis of melanoma: A retrospective cohort study in Xinjiang, China
Source: PLoS One. 2026 Sep 9;21(9):e0357383. doi: 10.1371/journal.pone.0357383 (PMC13557341; doi:10.1371/journal.pone.0357383)
Supplement: Supplementary file 7 [file pone.0357383.s007.docx]

S7 Table. Univariate and multivariate analysis of risk factors for OS in MM.

| Characteristics | Univariate analysis | | | Multivariate analysis | | |
| --- | --- | --- | --- | --- | --- | --- |
|  | HR | 95%CI | p value | HR | 95%CI | p value |
| Age |  |  |  |  |  |  |
| <60 |  | Reference |  |  |  |  |
| ≥60 | 1.78 | 0.77-4.16 | 0.180 |  |  |  |
| Gender |  |  |  |  |  |  |
| Female |  | Reference |  |  |  |  |
| Male | 0.74 | 0.30-1.86 | 0.524 |  |  |  |
| AJCC stage |  |  |  |  |  |  |
| 0&I&II |  | Reference |  |  |  |  |
| III&IV | 2.31 | 0.81-6.61 | 0.118 |  |  |  |
| Metastasis |  |  |  |  |  |  |
| Without |  | Reference |  |  | Reference |  |
| With | 3.04 | 1.21-7.66 | 0.018 | 1.43 | 0.41-4.97 | 0.574 |
| Surgical therapy |  |  |  |  |  |  |
| Without |  | Reference |  |  |  |  |
| With | 0.64 | 0.25-1.64 | 0.349 |  |  |  |
| Adjuvant therapy |  |  |  |  |  |  |
| Without |  | Reference |  |  |  |  |
| With | 0.50 | 0.21-1.16 | 0.108 |  |  |  |
| LDH，U/L |  |  |  |  |  |  |
| <250 |  | Reference |  |  | Reference |  |
| ≥250 | 8.69 | 2.02-37.46 | 0.004 | 7.05 | 1.41-35.31 | 0.018 |
| BMI |  |  |  |  |  |  |
| <25 |  | Reference |  |  |  |  |
| ≥25 | 0.88 | 0.36-2.12 | 0.767 |  |  |  |
| aCCI |  |  |  |  |  |  |
| <3 |  | Reference |  |  |  |  |
| ≥3 | 1.46 | 0.63-3.40 | 0.376 |  |  |  |

*OS* Overall survival, *MM* Mucosal melanoma, *HR* Hazard ratio, *CI* Confidence interval, *AJCC* American Joint Committee on Cancer, *LDH* Lactate dehydrogenase, *BMI* Body mass index, *aCCI* Age-adjusted Charlson Comorbidity Index
