# Supplementary material for: Clinicopathological characteristics and prognosis of melanoma: A retrospective cohort study in Xinjiang, China
Source: PLoS One. 2026 Sep 9;21(9):e0357383. doi: 10.1371/journal.pone.0357383 (PMC13557341; doi:10.1371/journal.pone.0357383)
Supplement: Supplementary file 8 [file pone.0357383.s008.docx]

S8 Table. Univariate and multivariate analysis of risk factors for DFS in MM.

| Characteristics | Univariate analysis | | | Multivariate analysis | | |
| --- | --- | --- | --- | --- | --- | --- |
|  | HR | 95%CI | p value | HR | 95%CI | p value |
| Age |  |  |  |  |  |  |
| <60 |  | Reference |  |  |  |  |
| ≥60 | 3.19 | 1.39-7.35 | 0.006 | 6.33 | 2.15-18.71 | 0.001 |
| Gender |  |  |  |  |  |  |
| Female |  | Reference |  |  |  |  |
| Male | 1.01 | 0.45-2.30 | 0.974 |  |  |  |
| AJCC stage |  |  |  |  |  |  |
| 0&I&II |  | Reference |  |  |  |  |
| III&IV | 1.99 | 0.74-5.34 | 0.171 |  |  |  |
| Metastasis |  |  |  |  |  |  |
| Without |  | Reference |  |  | Reference |  |
| With | 2.69 | 1.08-6.73 | 0.034 | 1.43 | 0.53-3.85 | 0.475 |
| Adjuvant therapy |  |  |  |  |  |  |
| Without |  | Reference |  |  |  |  |
| With | 0.69 | 0.31-1.55 | 0.368 |  |  |  |
| LDH，U/L |  |  |  |  |  |  |
| <250 |  | Reference |  |  | Reference |  |
| ≥250 | 3.08 | 0.84-11.26 | 0.090 | 4.76 | 1.15-19.72 | 0.032 |
| BMI |  |  |  |  |  |  |
| <25 |  | Reference |  |  |  |  |
| ≥25 | 1.15 | 0.49-2.66 | 0.750 |  |  |  |
| aCCI |  |  |  |  |  |  |
| <3 |  | Reference |  |  |  |  |
| ≥3 | 1.96 | 0.87-4.42 | 0.103 |  |  |  |

*DFS* Disease-free survival, *MM* mMucosal melanoma, *HR* Hazard ratio, *CI* Confidence interval, *AJCC* American Joint Committee on Cancer, *LDH* Lactate dehydrogenase, *BMI* Body mass index, *aCCI* Age-adjusted Charlson Comorbidity Index
