# Supplementary material for: Clinicopathological characteristics and prognosis of melanoma: A retrospective cohort study in Xinjiang, China
Source: PLoS One. 2026 Sep 9;21(9):e0357383. doi: 10.1371/journal.pone.0357383 (PMC13557341; doi:10.1371/journal.pone.0357383)
Supplement: Supplementary file 9 [file pone.0357383.s009.docx]

S9 Table. Univariate and multivariate analysis of risk factors for OS in MUP.

| Characteristics | Univariate analysis | | | Multivariate analysis | | |
| --- | --- | --- | --- | --- | --- | --- |
|  | HR | 95%CI | p value | HR | 95%CI | p value |
| Age |  |  |  |  |  |  |
| <60 |  | Reference |  |  | Reference |  |
| ≥60 | 3.53 | 1.27-9.78 | 0.015 | 3.26 | 0.37-28.69 | 0.287 |
| Gender |  |  |  |  |  |  |
| Female |  | Reference |  |  |  |  |
| Male | 2.36 | 0.78-7.18 | 0.129 |  |  |  |
| Distant metastasis |  |  |  |  |  |  |
| Without |  | Reference |  |  | Reference |  |
| With | 6.97 | 1.53-31.68 | 0.012 | 2.99 | 0.13-68.43 | 0.492 |
| Surgical therapy |  |  |  |  |  |  |
| Without |  | Reference |  |  |  |  |
| With | 0.52 | 0.20-1.32 | 0.166 |  |  |  |
| Adjuvant therapy |  |  |  |  |  |  |
| Without |  |  |  |  |  |  |
| With | 0.98 | 0.40-2.43 | 0.968 |  |  |  |
| LDH，U/L |  |  |  |  |  |  |
| <250 |  | Reference |  |  | Reference |  |
| ≥250 | 8.69 | 2.02-37.46 | 0.004 | 32.45 | 1.07-985.56 | 0.046 |
| BMI |  |  |  |  |  |  |
| <25 |  | Reference |  |  |  |  |
| ≥25 | 1.39 | 0.54-3.60 | 0.499 |  |  |  |
| aCCI |  |  |  |  |  |  |
| <3 |  | Reference |  |  | Reference |  |
| ≥3 | 4.18 | 1.42-12.29 | 0.009 | 8.68 | 0.67-112.49 | 0.098 |

*OS* Overall survival, *MUP* Melanoma of unknown primary, *HR* Hazard ratio, *CI* Confidence interval, *LDH* Lactate dehydrogenase, *BMI* Body mass index, *aCCI* Age-adjusted Charlson Comorbidity Index
