# Supplementary material for: Clinicopathological characteristics and prognosis of melanoma: A retrospective cohort study in Xinjiang, China
Source: PLoS One. 2026 Sep 9;21(9):e0357383. doi: 10.1371/journal.pone.0357383 (PMC13557341; doi:10.1371/journal.pone.0357383)
Supplement: Supplementary file 10 [file pone.0357383.s010.docx]

S10 Table. Univariate and multivariate analysis of risk factors for DFS in MUP.

| Characteristics | Univariate analysis | | | Multivariate analysis | | |
| --- | --- | --- | --- | --- | --- | --- |
|  | HR | 95%CI | p value | HR | 95%CI | p value |
| Age |  |  |  |  |  |  |
| <60 |  | Reference |  |  |  |  |
| ≥60 | 2.16 | 0.58-8.05 | 0.252 |  |  |  |
| Gender |  |  |  |  |  |  |
| Female |  | Reference |  |  |  |  |
| Male | 1.40 | 0.50-3.95 | 0.526 |  |  |  |
| Distant metastasis |  |  |  |  |  |  |
| Without |  | Reference |  |  | Reference |  |
| With | 6.94 | 1.48-32.61 | 0.014 | 2.50 | 0.41-15.32 | 0.323 |
| Adjuvant therapy |  |  |  |  |  |  |
| Without |  | Reference |  |  |  |  |
| With | 0.87 | 0.31-2.39 | 0.779 |  |  |  |
| LDH，U/L |  |  |  |  |  |  |
| <250 |  | Reference |  |  | Reference |  |
| ≥250 | 5.11 | 0.82-31.79 | 0.080 | 2.91 | 0.43-19.55 | 0.272 |
| BMI |  |  |  |  |  |  |
| <25 |  | Reference |  |  |  |  |
| ≥25 | 0.90 | 0.30-2.69 | 0.844 |  |  |  |
| aCCI |  |  |  |  |  |  |
| <3 |  | Reference |  |  |  |  |
| ≥3 | 1.82 | 0.40-8.36 | 0.441 |  |  |  |

*DFS* Disease-free survival, *MUP* Melanoma of unknown primary, *HR* Hazard ratio, *CI* Confidence interval, *LDH* Lactate dehydrogenase, *BMI* Body mass index, *aCCI* Age-adjusted Charlson Comorbidity Index
