# Supplementary material for: Clinicopathological characteristics and prognosis of melanoma: A retrospective cohort study in Xinjiang, China
Source: PLoS One. 2026 Sep 9;21(9):e0357383. doi: 10.1371/journal.pone.0357383 (PMC13557341; doi:10.1371/journal.pone.0357383)
Supplement: Supplementary file 11 [file pone.0357383.s011.docx]

| Model | Outcome | N | Events | C-index | LR χ² | LR p-value | AIC | BIC | Global PH χ² | Global PH p-value | PH Assumption Satisfied | Violations |
| --- | --- | --- | --- | --- | --- | --- | --- | --- | --- | --- | --- | --- |
| S1 Table | OS | 144 | 29 | 0.856 | 34.78 | 2.90e-05 | 205.98 | 229.74 | 0.965 | 0.3258 | Yes | None |
| S2 Table | DFS | 132 | 34 | 0.8327 | 37.65 | 4.00e-06 | 235.55 | 255.73 | 0.092 | 0.7611 | Yes | None |
| S3 Table | OS | 89 | 16 | 0.8858 | 21.66 | 0.0029 | 92.16 | 109.58 | 0.035 | 0.8508 | Yes | None |
| S4 Table | DFS | 74 | 17 | 0.8805 | 29.22 | 0.0001 | 101.29 | 117.42 | 0 | 0.984 | Yes | None |
| S5 Table | OS | 104 | 19 | 0.8449 | 23.55 | 0.0003 | 123.45 | 136.68 | 0.159 | 0.6897 | Yes | Breslow (p=0.047) |
| S6 Table | DFS | 84 | 21 | 0.8774 | 33.44 | 2.20e-05 | 125.48 | 142.49 | 0.496 | 0.4814 | Yes | None |
| S7 Table | OS | 47 | 15 | 0.6527 | 5.82 | 0.0544 | 87.68 | 91.38 | 0.537 | 0.4635 | Yes | None |
| S8 Table | DFS | 39 | 20 | 0.6227 | 3.81 | 0.2833 | 112.07 | 117.06 | 0.159 | 0.6899 | Yes | None |
| S9 Table | OS | 17 | 9 | N/A | N/A | N/A | N/A | N/A | N/A | N/A | N/A | Insufficient sample (n<20) |
| S10 Table | DFS | 13 | 9 | N/A | N/A | N/A | N/A | N/A | N/A | N/A | N/A | Insufficient sample (n<20) |

S11 Table. Cox regression model performance evaluation across all subgroups.
